# The influence of body morphology on changes in core temperature during exercise in an uncompensable environment

**DOI:** 10.1186/2046-7648-4-S1-A143

**Published:** 2015-09-14

**Authors:** Nicholas Ravanelli, Matthew Cramer, Pascal Imbeault, Ollie Jay

**Affiliations:** 1 School of Human Kinetics, University of Ottawa, Canada; 2 Thermal Ergonomics Laboratory, Faculty of Health Sciences, University of Sydney, Australia

## Introduction

Evidence demonstrates that for unbiased comparisons of changes in core temperature (ΔT_core_) between groups unmatched for body morphology, exercise should be performed using a fixed heat production (H_prod_) per unit mass in physiologically compensable environments [1]. In uncompensable conditions, it has been suggested that a fixed external workload is the primary determinant of ΔT_core _[2], however in addition to not accounting for differences in H_prod _relative to mass, such an approach excludes the influence of differences the surface area-to-mass ratio on the absolute maximum rate of evaporative heat loss (E_max_). We examined the best method for performing unbiased comparisons of ΔT_core _between groups unmatched for body morphology during exercise in an uncompensable environment.

## Methods

Six small (mean(SD) SM: 64.4(7.2) kg, 1.78(0.10) m^2^, 276(21) cm^2^.kg^-1^) and four large (LG: 94.2(7.2) kg, 2.19(0.09) m^2^, 233(8) cm^2^.kg^-1^) participants were recruited. E_max _for each participant was first assessed [3]. Participants then completed three trials, during which they cycled for 75 min at 35 °C, 70 % RH, at a target (i) absolute workload of 100 W, (ii) H_prod _of 6 W.kg^-1^, or (iii) H_prod _of 3 W.kg^-1 ^above E_max_.

## Results

**Figure 1:**
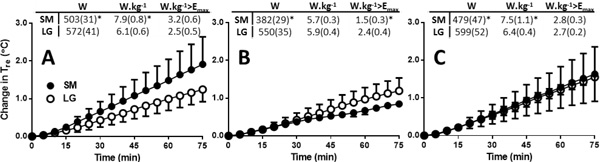
**The change in rectal temperature (Tre) during exercise at a fixed: external workload of 100 W (A), Hprod of 6 W.kg-1 (B), and Hprod of 3 W.kg-1 above Emax (C). SM small; LG large.** *Significantly different between groups within condition (P < 0.05).

E_max _at 35 °C, 70 % RH was similar between SM and LG in W.m^-2 ^(167 [27] vs. 146 [9] W.m^-2^), but lower in LG in W/kg (3.4 (0.2) vs. 4.6 (0.1) W.kg^-1^) by virtue of a difference in surface area-to-mass ratio. A systematically greater ΔT_re _was observed in the SM group at an external workload of 100 W (P = 0.036; Figure 1A); and in the LG group at an H_prod _of 6 W.kg^-1 ^(P < 0.001; Figure 1B). This systematic difference in ΔT_re _between SM and LG groups was abolished at a fixed H_prod _of 3 W.kg^-1 ^above E_max _(P = 0.999; Figure 1C).

## Discussion

Theoretically, ΔT_re _in an uncompensable environment should be determined by the rate of heat storage per unit mass, which is presently expressed as the difference between H_prod _and E_max _in W.kg^-1^. At a fixed absolute workload of 100 W, ΔT_re _and H_prod_-E_max _in W.kg^-1 ^were greater in SM. At a fixed H_prod _of 6 W.kg^-1^, ΔT_re _and H_prod_-E_max _in W.kg^-1 ^as greater in LG due to a smaller surface area-to-mass ratio. When H_prod_-E_max _in W.kg^-1 ^was fixed between SM and LG, ΔT_re _was the same despite a different H_prod _in W.kg^-1^.

## Conclusion

Preliminary results suggest that over a fixed exercise duration in an uncompensable environment, unbiased comparisons of ΔT_re _between groups/individuals of different body size (mass and BSA) may be best attained using an exercise intensity at a fixed H_prod_-E_max _in W.kg^-1^.
